# Book Review: Skin in the Game

**DOI:** 10.3389/fpsyg.2018.01640

**Published:** 2018-09-04

**Authors:** Jose D. Perezgonzalez

**Affiliations:** Business School, Massey University, Palmerston North, New Zealand

**Keywords:** science, epistemology, ethics, asymmetry, game theory

*Skin in the Game* is a book about asymmetries in life, in society, in history, in beliefs. Written by Nassim Nicholas Taleb, a former options trader turned probability thinker via his own skin in the game, the book is also about the consequences of such asymmetries. It explores four main topics in a somewhat haphazard manner, “according to how deep the author wants to go into a topic, not to make life easy for the critics to write reviews” (p. 44). The main topic is that of symmetry in human affairs, expanded into a second—related—topic of symmetry in information sharing in market-type transactions. A third topic is the exposition of a survival-bound definition of rationality. The fourth—and most overarching—topic is uncertainty and knowledge reliability, especially in regards to risk management in the marketplace, war, business, etc.

Taleb also uses the book to touch on science and academic life. Indeed, it would be fair to say that Taleb does not have a very good opinion of academics, whom he portrays, in general, as charlatans with an interventionist mindset and not much common sense; biased and blind to contradictions; immersed in an unethical academic game resembling an athletic contest of complicated scholarship to look good; submissive to, and dependable for, self-serving institutions, themselves gaming the system; and gagged by others' skin in the game; namely, the epitome of the intellectual yet idiot person. He is not very kind to “nondoers,” “bad middlemen,” and “full of baloney” book reviewers, either (p. 43–44).

But if we go past above remarks, we find that it is in the scientific realm that the book offers valuable insights to some of the epistemological and ethical aspects pestering contemporary science. The first insight is the uncertainty and knowledge unreliability of our models, especially applicable to those fields having to do with risk management: An insight of theoretical and statistical concern when modeling risk and risk taking, but also of concern when applying research knowledge to practice (also Stengers, 2018).

A second insight is about the distortion of symmetry and the agency problem of the current way of doing science. Protected in the cocoon of the Ivory Tower, the typical academic can research without having to pay for the consequences of what is published, transferring risk down to readers until a blow up, such as the current crisis of replicability (Open Science Collaboration, 2012). In so transferring risk within a system not designed to prevent it, both the academic and the system are unable to learn from the former's mistakes.

A third insight is that such distortion in symmetry seems to be compounded by asymmetric information sharing. Contemporary career progression in science not just depends on publishing research but on publishing with impact (i.e., on selling it and upping the ratings). Researchers hold all the information in regards to what methods to use, how to analyze data, and what to publish. Meanwhile, the readers—those “random suckers far away” to whom local ethics may not apply (p. 54)—are simply left with a pristine printout and a *caveat emptor* feeling about transparency. This asymmetric landscape resembles the “free rider dilemma” in game theory, albeit in an ever-expanding universe of possibilities where asymmetry by risk transfer can easily survive and prosper (also Smaldino and McElreath, 2016).

A last insight is the test of time on rationality, where science, including the current peer-review system, is not Lindy proof—albeit this idea brings with it a contradiction not resolved by Taleb: That “bad” science and the academic enterprise he so thoroughly criticizes ought to be considered “rational,” conditioned on them having survived thus far. And even if the contradiction could be resolved, it may still avail of his own disclaimer on page 27: That much of contemporary science can be considered “a harmless opinion…or a pronouncement…[not immediately suitable for] decision making.”

Interspersed within the book there are also potential solutions to above state-of-affairs. A first solution is a systemic one: Let people alone under a good structure that may ensure the overall good performance of the system even if individual performance varies. It, perhaps, is a bit *risqué* to call such structure an “invisible hand” but it could certainly work as a point of reference onto which researchers could fold back from time to time. The paramount structure is continual education in the tools of research. That is, educate scientists, and allow them to re-educate themselves, in how to use methods and statistics well, and in how to interpret probability appropriately. For example, use fat-tail analyses (a.k.a., extreme value analyses) when dealing with risk management—a fundamental idea already introduced in Taleb's previous works Taleb (2001, 2007, 2012).

A second solution is social and, necessarily, of more limited reach: the minority rule. We can already see such minority rule in reactions to the replicability crisis, with some journals banning the use of *p*-values (e.g., Trafimow and Marks, 2015), the jealousy of pre-registration proponents (e.g., Nosek et al., 2018), and Bayes Factor proponents (e.g., Morey et al., 2016), etc. Joining those “minority” movements that we agree are improving science may help bring about a change proportionally greater than that expected from their size alone.

A third solution is an ethical one for the individual researcher: to put some soul in the game. That is, to do quality work for “honor as an existential commitment” (p. 33), and to accept the consequences that it may not bring about as much personal benefit than cutting corners do in the manner inferences are substantiated and research is published.

In summary: Science is not so much a complex as a complicated system, with multiple agents vying for results that contradict not only those of other agents but science's own purpose, in general. No single solution will repair such system. Taleb's book pitches the complication, asserts the difficulty, and proposes solutions that may help patch it: time, understanding, and commitment. In a few words: skin in the game.

## Author contributions

The author confirms being the sole contributor of this work and approved it for publication.

### Conflict of interest statement

The author declares that the research was conducted in the absence of any commercial or financial relationships that could be construed as a potential conflict of interest.
